# Addictive Use of Smartphones and Mental Disorders in University Students

**Published:** 2020-04

**Authors:** Seyyed Salman Alavi, Maryam Ghanizadeh, Malihe Farahani, Fereshteh Jannatifard, Sudeh Esmaili Alamuti, Mohammad Reza Mohammadi

**Affiliations:** 1 Psychiatry and Psychology Research Center, Tehran University of Medical Sciences, Tehran, Iran.; 2 Kerman University of Medical Sciences, Kerman, Iran.; 3 Department of Psychology, Najafabad Branch, Islamic Azad University, Najafabad, Iran.; 4 Ministry of Education, Tehran, Iran.; 5 Department of Psychology, Allame Tabataba’i University, Tehran, Iran.

**Keywords:** *Mental Disorders*, *Smart Mobile Phone Addiction*, *Student*

## Abstract

**Objective:** Despite the awareness of smartphone addiction, low findings and lack of validated standards have led to insufficient information in this area. This study aimed to examine the relationship between mobile phone dependency and mental disorders in students in Iran, while controlling for the impact of gender, age, educational levels, and marital status.

**Method** **:** In this cross-sectional study, a sample of 1400 university students (445 males and 955 females) aged 18-35 years were selected from 3 cities of Tehran, Isfahan and Karaj. The participants filled out a set of questionnaires: Cell Phone Dependency Questionnaire (CPDQ), Millon Multiaxial Clinical Inventory, and participated in interviews. Data were analyzed using multiple logistic regression method via SPSS-22 software.

**Results: **In terms of controlling the demographic variables, the results revealed that bipolar disorder, depression, anxiety, somatization, dependent personality disorder, and compulsive personality disorder could increase the possibility of mobile phone addiction by 4.2, 4.2, 1.2, 2.8, 3.1, and 3.2 folds, respectively (P < 0.05). However, other disorders and demographic characteristics did not have any significant effects on the equation.

**Conclusion: **The results can help better understand the relationship between psychological syndromes and smartphone addiction and can also facilitate further studies in this field. In addition, those students with smartphone addiction should be provided with different preventative strategies. Moreover, a growing range of stimulating applications may trigger the risk of addiction.

Constant internet access (1) has become highly pervasive in recent years (2-4). According to Nielsen (2012), 47% of the world’s social media use is performed via mobile devices, he also found that 90% of American adults (3), over 80% of Australians, 62% of Asians, and more than 80% in some Asian countries, including Korea, China, and Japan, have mobile phones (5).

 Smartphones are not only used for talking (6); they have such features as games, texting, internet use (3), social groups (6), safety, and security (7). 

However, using them in socially inappropriate or physically dangerous in some situations (eg, texting while driving) (3) may lead to injury and death (6, 8-11).

Classrooms (12) and social environments are disrupted when smartphones are used at inappropriate times (6) and may lead to academic deﬁcits (9, 13-15). Mobile phones are indispensable for many young adults, but such devices may negatively affect their mental health and can even cause serious physiological reactions (16).

Some people experience excessive or uncontrolled use of smartphones (2, 3, 17). 

For the ‎ first time, Peele (18) stated that addiction is not just related to substance or chemical addiction (3, 6, 19-23), and what makes people become addicted to any particular behavior may be ‎ considered as an addiction (22), which could result in functional impairment (24).

Mobile phone addiction shares similarities to other behavioral dependency including video game addiction, compulsive shopping or pathological gambling, (3, 25).

Technological addictions and the aforementioned list of cognitive symptoms share common characteristics with substance addiction (3) and include 4 components of tolerance, withdrawal (3, 6), compulsive symptoms, and functional impairment (2). Like any other addiction, the denial of the addiction and discontinuing therapy are also very common in the case of patients (21, 26).

Chen (2004) reported that 25% of American college students diagnosed as addicted to mobile phone (27). In addition, 21.4% of Iranian adolescents and 27.4% of adolescents in Hong Kong have been categorized as cell phone addicts (28-30). 

Some people cannot regulate their excessive use of mobile phones (3). An increase in the frequency of mobile phone use causes mental health problems among university students (21). Studies have shown that compulsive use of smartphones may lead to psychological disorders (9, 20, 31-35) and different types of psychopathology, including depression (4, 9, 20, 26, 36, 37) and anxiety (4, 9, 19, 20, 26, 34, 36-38) in college students (9, 24). Khang et al (2012) identiﬁed compulsive anxiety as a factor in cell phone addiction (39). Steelman et al (2012) found that dangerous mobile phone use is similar to OCD behaviors, reﬂecting anxiety and stress in attempting to manage responsibilities (39). Some studies have found that social media use may predict symptoms for a range of personality and mood disorders (eg, bipolar-mania)(40). Problematic smartphone use may create emotional dysfunction or mental disorders (15), which can especially be associated with PTSD severity (41).

Students’ increased mobile phone use may negatively impact sleep disturbance (3, 9, 30, 34, 36-39), financial problems (9, 15, 26, 36, 42), and academic performance (9, 15, 36, 42), and concentration difﬁculties (43).

People with higher depression severity prefer to overuse technology (44). Moreover, it has been suggested that individuals who experience social anxiety tend to be more compulsive smartphone users (1, 33).The relationship between depression and cell phone addiction is a critical issue because such symptoms may lead to substance abuse (45), school failure (46), and even suicide (47, 48).

 Studies revealed that females are more dependent on smartphones than males (24, 26, 42, 49). Some studies found that social status affected smartphone addiction and single participants scored higher than married participants on smartphone addiction (42). However, associations have been revealed between increased smartphones use and speciﬁc types of psychopathology (44). 

Despite the fact that more and more studies have surveyed the effect of psychological illnesses, including anxiety and depression, on mobile phone addiction, very few studies have focused on the impact of mental disorders on cell phone addiction based on psychiatric interview. 

Therefore, the present study was conducted to assess the following hypothesis: Is there any relationship between mental disorders and mobile phone addiction? Or in other words, can mental disorders predict mobile phone addiction in students? The purpose was to define factors that influence smart phone addiction based on mental disorders. Iran is not immune to negative effects of virtual spaces. Thus, designing appropriate models to describe factors affecting mobile phone addiction is of paramount importance.

The purpose of this study was to examine the mobile overuse related to psychological disorders on clinical symptoms of mood disorders (eg, major depression, dysthymia and mania, anxiety, PTSD, somatization, dependence, narcissism, antisocial personality disorder, OCD, paranoia, histrionic personality disorder, and schizoid personality disorder) and to focuse on mental health correlates of smartphone use in a sample of students because the likelihood of smartphone addiction is increasing among students (21, 42). Also, research showed young people are more vulnerable to excessive phone use, thus, become phone-dependent (28).

## Materials and Methods

***Procedure and Sample***

 This descriptive and analytical study aimed to determine the relationship between mental disorders and excessive smart mobile phone use. Participants were 18-35-year-old students (N = 1400). The sample included 955 (68.2%) females and 445 males (31.8%). Exclusion criteria were as follow: participants with severe physical problems or apparent disability and those who did not use smart mobile phones. This research and its study protocol were supported and approved by Tehran University of Medical Sciences and Health Services (grant number: 34781).

Participants were selected from various universities in Tehran, Isfahan, and Karaj. The sociodemographic questionnaires were filled as per the information given by the participants. Moreover, Mobile Phone Addiction Questionnaire, Millon Questionnaire, SCID I, and screening interview for smartphone addiction disorders were conducted for individual screening. Semi-structured interviews were performed by 2 specially trained psychologists who held a PhD degree in clinical psychology. They had successfully passed an internship course in clinical interviewing, symptomatology, and diagnosis of disorders. The sample comprised of more than 1400 students; however, about 150 questionnaires were not completed due to students’ lack of time.

***Measures***

This study included 4 types of instruments: (1) the Structured Clinical Interview to diagnose mental disorders (SCID), (2) Cellular Phone Dependency Questionnaire (CPDQ) (3), Semi- structured interview to diagnose mobile phone addiction (4), and a questionnaire to diagnose personality disorders (Millon Multiaxial Clinical Inventory). In addition, demographic data, including age, gender, education level, marital status, time of mobile usage, and causes of mobile phone use, were collected.

**1)  Millon Multiaxial Clinical Inventory:** The MCMI-III is a psychological tool that can be utilized to provide information on the clinical symptoms of psychological disorders. It includes 175 items and yields data on a variety of Axis I mood disorders and Axis II personality disorders (50). For this study, only a subset of 9 MCMI subscales were selected, representing those disorders previously linked to smartphone inﬂuences: 3 personality disorder scales (dependent, compulsive and avoidant) and 4 mental disorder scales (bipolar, depression, OCD, anxiety).

The MCMI-III includes 2 additional scales, validity and disclosure, which were used to eliminate 11 participants with invalid scores. Participants’ raw scores were converted to base rate scores for use in all analyses. The diagnostic validity of this test in all scales ranges from 0.58 to 0.83 (50).

**2)  CPDQ (Cell Phone Dependency Questionnaire):** The CPDQ, a 20-item self-report questionnaire, was first used by Toda et al (2004). The participants responded to each item on a 5-point Likert spectrum scale. A higher score illustrated a stronger tendency toward mobile phone dependency. Toda et al (2004) reported that the questionnaire’s reliability coefficient for female university students in Japan was 0.86, and its internal consistency was confirmed (51).

Alavi et al (2014) reported that the CPDQ confirmed to be a valid questionnaire to, measure the extent of problems caused by the misuse of mobile phones in Iranian society. They also found that the CPDQ has 3 factors: salience, overusing mobile phones, and compulsive use of SMS. Internal consistency of the CPDQ was 0.88 (Cronbach's alpha of the factors was 0.85, 0.70, and 0.76, respectively) (52).

**3)  Structured Clinical Interview for DSM-IV Disorders (SCID-I):** Clinical psychiatrists conducted clinical interviews to diagnose any mental disorder in samples. The SCID is a diagnostic exam used to determine mental disorders (SCID-I). It was assessed by independent, trained, and supervised clinicians, and it is recommended as the gold standard tool for diagnosing mental disorders (53). In another study, Duarte-Guerra et al (2015) reported that the interrater reliability for SCID-I lifetime disorders was determined as kappa coefficient of 0.81 in a random sample of 15 patients (54). Also, kappa coefficient in Iranian research was calculated to be 0.55 for SCID-I lifetime disorders (55).

***4)*** **Semi-structured Interview Based on DSM-IV-TR** ***Criteria for ICD-NOS: ***This interview was performed by clinical psychologists trained for the diagnosis of impulse control disorder (ICD) in general and mobile addiction disorder in particular. 

***5)*** **Demographic Questionnaire:** A background data sheet was designed by the principal investigator to collect sociodemographic details of the participants, including gender, age, socioeconomic status, education level, marital status, and cell phone use.

***Statistical Analysis***

 Descriptive statistics were used to demonstrate demographic data. Chi-square was calculated to assess the significance of the associations among the demographic variables. Moreover, effective psychological disorders that contributed to smartphone addiction were determined using logistic regression analysis. Also, p value less than 0.05 was considered as statistically significant.

## Results

The mean and standard deviation of age in the sample was 25.17 ± 4.5 years, and age distribution was 18 to 35 years. The sample included 955 (68.2%) females and 445 males (31.8%). Of the participants, 924 were single (66%) and 476 were married (34%). No differences were found in the demographic information of the samples (P value > 0.05) (Table 1).

According to the results, female students tended to use mobile phones more frequently than males. The risk of smartphone addiction was about 1.2 times more in females than in males. In addition, the findings revealed that the odds of smartphone addiction in singles were about 1.5 times more than the married. The results indicated that among disorders, dependent personality disorder was 3.1 folds more likely to increase the probability of mobile addiction. Moreover, it was also found that compulsive personality disorders increased the chance of smartphone addiction (negative effect, OR = 3.2). However, other personality disorders did not seem to have a significant effect on the odds of mobile dependency (Table 2).

The results revealed that bipolar spectrum disorders increased the risk of smartphone addiction by 4.2 folds. In addition, with regards to mood disorders, particularly depressive disorder, the results showed that this disorder increased the risk of mobile phone addiction by 4.2 folds. 

Furthermore, it was found that anxiety increased the probability of smartphone addiction by 1.2 folds (negative effect). Based on the multiple logistic regression interpretations (multivariable analysis), somatization had an impact on the risk of mobile addiction (OR = 2.8) (Table 2).

Some mental illnesses such as PTSD and eating disorders, including bulimia or anorexia, narcissism personality disorders and other sociodemographic variables, such as average time used on mobile phones per day, were not significant parameters in the equation. Moreover, because none of the samples cited any delusion or hallucination, epilepsy, hypomania, panic disorder, fugue, or other mental problems, these disorders were not entered in the equation.

**Table 1:** Demographic Information of the Participants of Mobile Addiction According to Questionnaire and Interview)

**Demographic Properties**		**Diagnosis of Mobile Addiction**		
		**No**	**Yes**	**P-value**
University	University of Technology	100(10.5%)	46(10.2%)	0.06
	National University	213(22.5%)	66(14.6%)	
	University of Medical Sciences	31(3.3%)	7(1.5%)	
	Islamic Azad University	604(63.7%)	333(73.7%)	
Sex	Female	649(68.5%)	306(67.7%)	0.41
	Male	299(31.5%)	146(32.3%)	
Education	Undergraduate& Bachelor	637(67.2)	343(75.9%)	0.18
	MSc or PhD	311(32.8%)	109(24.1%)	
Marital status	Single	617(65.1%)	307(67.9%)	0. 16
	Married	331(34.9%)	145(32.1%)	
Age [year]	Mean±SD	25.17±4.4	25.19±4.7	0.94

**Table 2:** Odds Ratios [95% CI] for Mobile Addiction in University Students (n = 1400)

**Variables**	**Univariable Analysis**			**Multivariable Analysis**		
	**OR(crude)**	**CI (95%)**	**P-value**	**OR(adjusted)**	**CI (95%)**	**P-value**
Age	1.1	1.02-1.12	0.002	1.3	0.83-2.1	0.22
Sex [female VS Male]	1.2	0.78-1.7	0.13	1.2*	1.7-2.8	0.04
Marital Status [Single VS Married]	1.7	1.1-2.5	0.012	1.5*	1.9-2.5	0.05
Avoidant Personality Disorder	7.6	0.95-61	0.056	2.2	0.013-1.2	0.07
Dependent Personality Disorder	7.4	0,92-60	0.06	3.1*	1.38-6.92	0.006
Compulsive Personality Disorder	1.3	1.05-1.8	0.05	3.2*	1.4-6.7	0.003
Bipolar disorders	1.5	045-5.0	0.2	4.2*	1.27-14.1	0.01
PTSD	0.36	0.11-1.11	0.07	0.25	0.02-2.4	0.23
Cychlotymia	0.35	0.12-0.99	0.05	0.42	0.13-1.3	0.14
Depression	5.7	0.66-49	0.11	4.2*	4.6-39.2	0.01
Panic Disorder	0.31	0.11-0.86	0.02	0.45	0.14-1.4	0.18
OCD	0.52	0.33-0.81	0.004	049	0.29-8.3	0.08
Anxiety	1.8	0.68-4.76	0.2	1.2*	3.8-6.4	0.007
Somatization	1.7	1.2-2.7	0.015	2.8*	6.8-11.9	0.014
Anorexia	0.52	0.27-0.98	0.05	0.73	0.31-1.6	0.46
Bulimia	0.42	0.18-0.97	0.47	0.49	0.18-1.3	0.15

## Discussion

The aim of this study was to evaluate smartphone overuse and its association with psychiatric disorders among students aged 18 to 35 years in Iran. Our research hypothesis was based on the assumption that some mental illnesses would create mobile phone addiction. The results of this study provided fundamental information that could contribute to the diagnosis, treatment, and prevention of mobile phone addiction among adolescents.

According to the results, female students tend to use mobile phones more frequently than males. The risk of smartphone addiction was about 1.2 times more in women than in men. The results of the present study are more in line with other studies, indicating a significant relationship between female gender and mobile phone addiction.

Valkenburg and Peter (2007) reported that girls were significantly more socially anxious and closer to friends than were boys (56). Randler et al (2016) mentioned that gender is an crucial predictor for cell phone addiction and that girls are susceptible to become addicted. According to their findings, girls had higher scores than boys in cell phone addiction (57). Similarly, Otero-Lopez and Villardefrancos (2014) reported that there is a more possibility that females become compulsive buyers in the general population sample (58). Also, some studies indicated that women have higher rates of dependence and problematic use of mobile phone than males (59-61). Despite these findings, De-Sola et al (2017) reported that being male, having a lower education level, using a mobile phone for long hours, and age 35 years and to a lesser extent, up to 45 years old, increase the probability of addictive use of mobile phones (62). The inconsistency between our results and those of the above-cited studies could be due to different samples, personality types, and the effect of other variables, such as age and environmental factors. Moreover, these differences in findings may also be due to cultural variation in the use of mobile phones.

The difference between males and females in using smartphones is based on the time spent on the phone rather than its use. Females spend more time using their smartphones than males, which leads to developing intense and close social relationships, whereas males use their time in a more practical and instrumental way.

The results revealed that bipolar spectrum disorders increase the risk of smartphone addiction by 4.2 folds. In addition, with regards to mood disorders, particularly depressive disorder, the results revealed that this disorder increased the risk of mobile phone addiction by 4.2 folds. A previous research showed that a mobile phone can be used to avoid unpleasant moods (63). Mitra and Rangaswamy (2019) revealed that there is a correlation between using social media addiction and depression. Das A (2017) argued that the use of information technology may help avoid negative emotions (64). This has been mentioned in another study (65, 66). Abbasi et al (2016) reported that prevalence of addictive behaviors among bipolar patients is considered to be a serious health problem; moreover, they found a significant positive relationship between behavioral addictions, such as mobile or television addiction, with bipolar disorder (67). Many studies have emphasized the comorbidity of addictive behaviors in patients with bipolar disorders (68).

Martinotti et al found that higher levels of addictive behaviors are observed in patients with mental disorders, such as schizophrenia and bipolar patients (69). Furthermore, various researches have reported that the rate of drug abuse, smoking, and addictive behavior is higher in bipolar patients than in other patients (70-72). Thus, it can be inferred that patients with bipolar disorder use cell phones for mood alternation and as pleasure stimulants; they also use substances to decrease unfavorable stimulants and negative emotions. Findings indicate that inhibition systems and behavioral activation have a serious role in the exacerbation and incidence of addictive behaviors. Therefore, preventive programs seem to be of paramount importance.

The findings of our study showed that anxiety also increased the probability of smartphone addiction by 1.2 folds (positive effect). Also, the results on anxiety are highly compatible with those of previous studies. In Weidman et al (2012) study, individuals with social anxiety reported a greater feeling of comfort and self-disclosure when socializing online compared to face-to-face communication (73). Jafarkarimi et al (2016) indicated that virtual spaces, such as mobile phone or internet, are associated with depression, anxiety, dependency, or addiction (66,74). Demirci, Akgonul, and Akpinar (2015) revealed that anxiety, depression, and daytime dysfunction were higher in the group who overused smartphones than in the group with low smartphone use. Positive and robust correlations were found among addiction to smartphones and depression, anxiety levels, and some sleep quality scores (24). In a study performed on mobile phone users, it was indicated that anxiety, trait anxiety, and depression were higher in the group who overused smartphones than in the group with normal mobile use (75).

It can be concluded that anxiety or low self-esteem may be correlated with cell phone addiction. Such overuse of smartphones may lead to anxiety or depression, which can in turn result in mental problems. Therefore, adolescents with high anxiety/depression should be carefully monitored for mobile phone addiction. Moreover, age, gender, anxiety, and depression should be considered as the determinants of smart phone addiction scale score in the linear regression model.

Also, adolescents, especially students, were unaware of the amount of time they spent on their smartphones and its effects on their mental health. These results may be due to the association between low self-esteem and a feeling of social inadequacy. One acceptable reason for our results on anxiety is that some people overuse smartphones to escape from unpleasant feelings such as anxiety.

Based on the results of multivariable analysis, somatization had an impact on the risk of mobile addiction. Cerutti et al reported an impact of excessive internet and mobile use, which ranged from different types of headaches to other somatic symptoms (76). Tavakolizadeh et al (2014) also observed a coexistence association between mental health problems including depression, anxiety, somatization and excessive mobile phone use (77). Students with mobile phone disorders are more likely to experience physical symptoms. Thus, we suggest conducting further studies to confirm the results through clinical interviews. Therefore, interpretation of the findings should be made with caution. When a person is obsessed with an object, substance, or activity, he/she engages in a behavior even though it causes harm, such as poor work, physical problems, or study performance, or problems with family and friends (77). Setting standards for smartphone use may help prevent its potential harmful health effects (77). Al Barashdi et al (2016) suggested that psychoeducational intervention has combined traditional cognitive-behavioral relapse prevention strategies with meditation training and mindful movement and can be used to help students with smartphone addiction (77). Also, treatment of many disorders, especially depression, which is more prevalent than other disorders in adolescents (78), can be effective in treating behavioral disorders such as addiction to mobility.

## Limitation

This study had a few limitations. First, data were self-reported, as Nourthrup [1996] (79) has mentioned, this might have resulted in the overestimation. Self-reports rely on the respondents' truthfulness and self-perception, and therefore their reliability and validity may be limited (80); thus, participants may exaggerate their cellphone use. Second, the sample was drawn from Tehran and Karaj, which do not represent all cities of Iran. Third, the MCMI-III, which generated the psychological disorder signs, has been designed as a tool for individual adults who are using or seeking mental health or psychiatric services, and it has not been intended for the general population. However, other studies have reported using this measure with a nonclinical population, and there is no reason to believe that the measure would systematically distort the results such that could impact the results of the hypothesis testing. Future studies can focus on describing the psychological correlates among students with smartphone addiction problems using longitudinal methods. Also, with regards to future research, the findings of this study could extend to draw preventive, protective, and therapeutic programs for smartphone addiction.

## Conclusion

The findings revealed a strong association between some psychiatric disorders, including depression, anxiety, bipolar, dependent personality disorder, compulsive personality disorder, and somatization, with addiction to mobile phones. Also, it was found that depression, anxiety, and bipolar disorders can significantly predict addiction to smart phones in college students. Adolescent girls appear to use smartphones excessively. In sum, assessing the relationship between smartphone addiction and mental disorders in students is highly recommended. Such assessments can be useful to consult centers and help them reduce the rate of addiction to smart phones symptoms through considering variables and related treatments.
